# Racemic 4-(4-*tert*-butyl­phen­yl)-2,6-dimethyl­cyclo­hex-3-enecarboxylic acid

**DOI:** 10.1107/S1600536808003309

**Published:** 2008-02-06

**Authors:** Songwen Xie, Caryn R. O’Hearn, Paul D. Robinson

**Affiliations:** a Department of Natural, Information, and Mathematical Sciences, Indiana University Kokomo, Kokomo, IN 46904-9003, USA; b Department of Geology, Southern Illinois University at Carbondale, Carbondale, IL 62901-4324, USA

## Abstract

The chirality of the title compound, C_19_H_26_O_2_, is solely generated by the presence of the double bond in the cyclo­hexene ring. This compound was synthesized to study the inter­action of the two enanti­omers in the solid state. The resultant racemate is made up of carboxylic acid *RS* dimers. Inter­molecular O—H⋯O hydrogen bonds produce centrosymmetric *R*_2_^2^(8) rings which dimerize the two chiral enanti­omers through their carboxyl groups.

## Related literature

In similar compounds previously reported (Xie *et al.*, 2002 ▶, 2007*a* ▶), the racemates also consist of carboxylic acid *RS* dimers. For related literature, see: Xie *et al.* (2007*b* ▶, 2004 ▶); Bernstein *et al.* (1995 ▶).
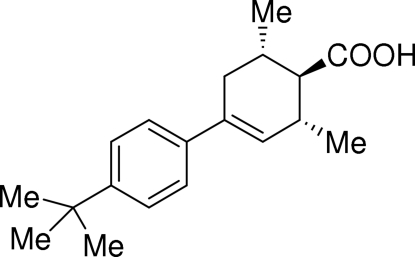


## Experimental

### 

#### Crystal data

C_19_H_26_O_2_
*M* *_r_* = 286.40
Monoclinic, 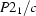

*a* = 24.818 (4) Å
*b* = 9.4674 (18) Å
*c* = 7.0105 (12) Å
β = 95.799 (5)°
*V* = 1638.8 (5) Å^3^
*Z* = 4
Mo *K*α radiation
μ = 0.07 mm^−1^
*T* = 100 (2) K
0.36 × 0.29 × 0.09 mm

#### Data collection

Bruker Kappa APEXII CCD diffractometer
Absorption correction: multi-scan (*SADABS*; Bruker, 2005 ▶) *T*_min_ = 0.793, *T*_max_ = 0.993
24559 measured reflections
2912 independent reflections
2230 reflections with *I* > 2σ(*I*)
*R*_int_ = 0.062

#### Refinement

*R*[*F* ^2^ > 2σ(*F* ^2^)] = 0.085
*wR*(*F* ^2^) = 0.244
*S* = 1.15
2912 reflections
196 parameters
H-atom parameters constrained
Δρ_max_ = 0.36 e Å^−3^
Δρ_min_ = −0.34 e Å^−3^

### 

Data collection: *APEX2* (Bruker, 2005 ▶); cell refinement: *SAINT* (Bruker, 2005 ▶); data reduction: *SAINT* and *SADABS* (Bruker, 2005 ▶); program(s) used to solve structure: *SIR92* (Altomare *et al.*, 1994 ▶); program(s) used to refine structure: *LS* in *TEXSAN* (Molecular Structure Corporation, 1997 ▶) and *SHELXL97* (Sheldrick, 2008 ▶); molecular graphics: *PLATON* (Spek, 2003 ▶); software used to prepare material for publication: *SHELXL97* and *PLATON*.

## Supplementary Material

Crystal structure: contains datablocks global, I. DOI: 10.1107/S1600536808003309/om2210sup1.cif

Structure factors: contains datablocks I. DOI: 10.1107/S1600536808003309/om2210Isup2.hkl

Additional supplementary materials:  crystallographic information; 3D view; checkCIF report

## Figures and Tables

**Table 1:** Hydrogen-bond geometry (Å, °)

*D*—H⋯*A*	*D*—H	H⋯*A*	*D*⋯*A*	*D*—H⋯*A*
O2—H2⋯O1^i^	0.82	1.88	2.702 (4)	175

Symmetry code: (i) 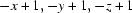.

**Fig. 1.**
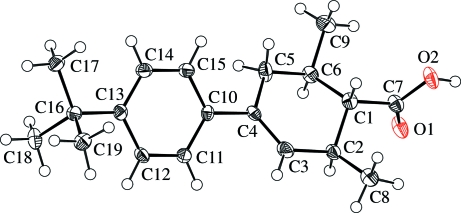
The molecular structure and atom numbering scheme, with displacement ellipsoids drawn at the 50% probablilty level.

**Fig. 2.**
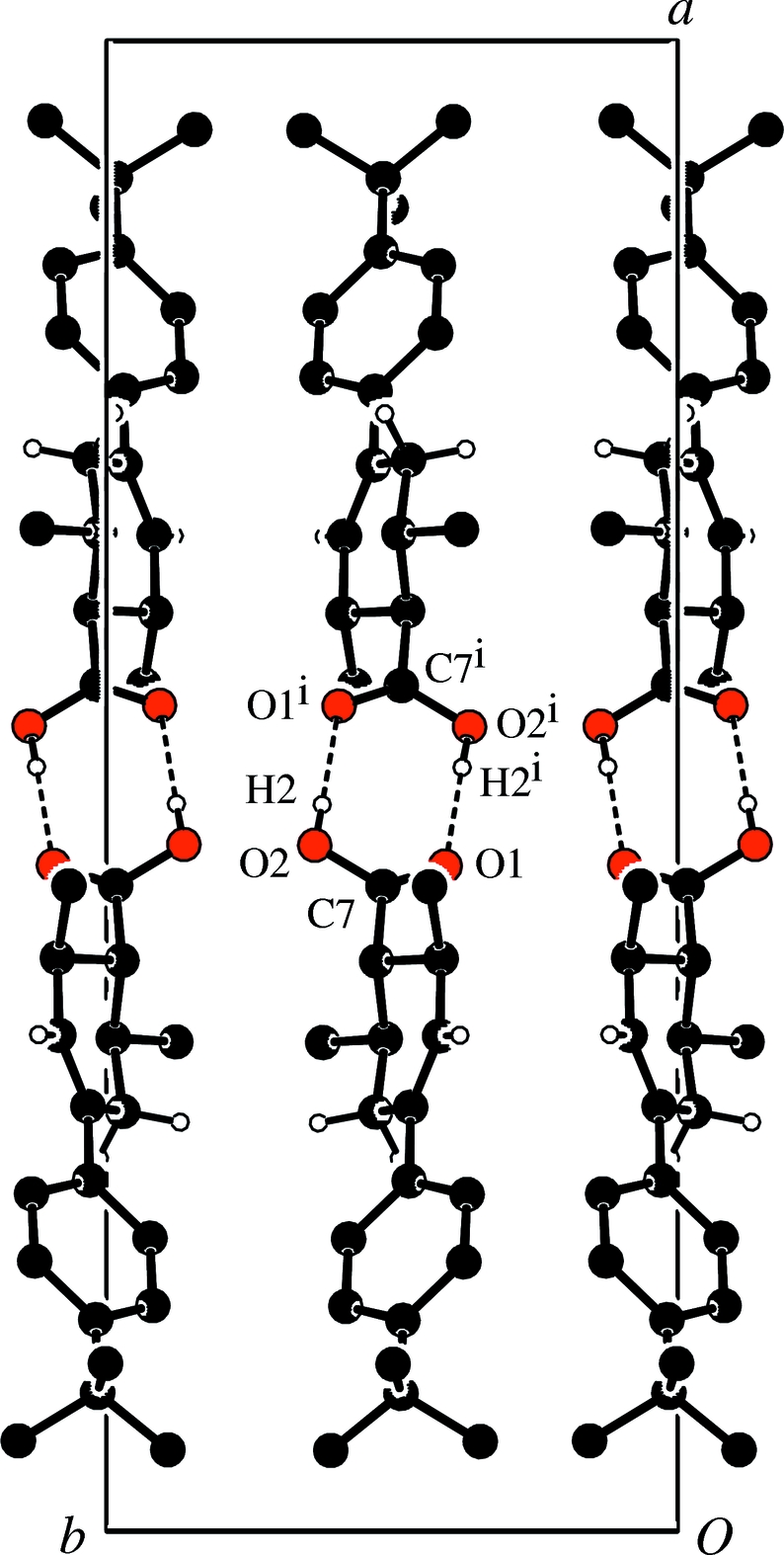
Molecular packing and hydrogen bonding as viewed down [001]. Dashed lines represent hydrogen bonds. Most H atoms not involved in hydrogen bonding have been omitted to improve clarity. [Symmetry code: (i) -x + 1, -y + 1, -z + 1.]

## supplementary crystallographic information

### Comment

The title carboxylic acid, the structure of whose single enantiomer is unknown, was prepared to study the interaction of the two enantiomers in the solid state. We have previously reported the structure of its precursor, which is achiral and also forms hydrogen-bonded dimers (Xie *et al.*, 2007*b*). The chirality of the title compound is solely generated by the presence of the double bond in the cyclohexene ring (Xie *et al.*, 2004). The resultant racemate is made up of carboxylic acid *RS* dimers. The structure and atom numbering are shown in Fig. 1, which illustrates the half-chair conformation of the cyclohexene ring. The torsion angles involving atoms C2, C3, C4, C5, and C6 are all near 180°, as are those involving atoms C8, C2, C1, C6, and C9. The carboxyl group is almost perpendicular to the cyclohexene ring with an angle of 81.6 (5) ° between the O1—C7—O2 plane and the C1—C6 ring. The double bond between C3—C4 is not fully conjugated as shown by the C3—C4—C5 plane to benzene ring angle of 30.4 (5) °.

Fig. 2 shows the hydrogen bonding scheme and molecular packing. Atom O2 acts as a donor in an intermolecular hydrogen bond to atom O1. Inversion of this interaction across (1/2, 1/2, 1/2) produces an *R*_2_^2^(8) ring (Bernstein *et al.*, 1995), thus creating a hydrogen-bonded *RS* dimer. There is no evidence to suggest that weak directional interactions interconnect the dimers. Hydrogen bond geometry is given in Table 1.

### Experimental

The title carboxylic acid was synthesized following a similar method previously reported by Xie *et al.*, 2002. Purified compound was recrystallized from hexane-ethyl acetate as colorless crystals (m.p. 467–468 K).

### Refinement

The rotational orientations of the methyl H atoms were refined by the circular Fourier method available in *SHELXL97* (Sheldrick, 2008); the hydroxyl H atom position was determined in a similar manner. All H atoms were treated as riding with C/O—H distances ranging from 0.82 to 0.98 Å and *U*_iso_(H) values equal to 1.5 (hydroxyl and methyl H atoms) or 1.2 times (all other H atoms) *U*_eq_ of the parent atom. The crystal diffracted poorly resulting in a relatively low accuracy refinement.

### Crystal data

**Table:**

C_19_H_26_O_2_	*F*_000_ = 624
*M**_r_* = 286.40	*D*_x_ = 1.161 Mg m^−^^3^
Monoclinic, *P*2_1_/*c*	Melting point = 467–468 K
Hall symbol: -P 2ybc	Mo *K*α radiation λ = 0.71069 Å
*a* = 24.818 (4) Å	Cell parameters from 5539 reflections
*b* = 9.4674 (18) Å	θ = 3.3–25.0º
*c* = 7.0105 (12) Å	µ = 0.07 mm^−^^1^
β = 95.799 (5)º	*T* = 100 (2) K
*V* = 1638.8 (5) Å^3^	Plate, colorless
*Z* = 4	0.36 × 0.29 × 0.09 mm

### Data collection

**Table:**

Bruker Kappa-APEXII CCD diffractometer	2912 independent reflections
Radiation source: X-ray tube	2230 reflections with *I* > 2σ(*I*)
Monochromator: graphite	*R*_int_ = 0.062
*T* = 100(2) K	θ_max_ = 25.1º
φ and ω scans	θ_min_ = 1.6º
Absorption correction: multi-scan(SADABS; Bruker, 2005)	*h* = −29→29
*T*_min_ = 0.793, *T*_max_ = 0.993	*k* = −11→11
24559 measured reflections	*l* = −8→8

### Refinement

**Table:**

Refinement on *F*^2^	Secondary atom site location: difference Fourier map
Least-squares matrix: full	Hydrogen site location: inferred from neighbouring sites
*R*[*F*^2^ > 2σ(*F*^2^)] = 0.085	H-atom parameters constrained
*wR*(*F*^2^) = 0.244	*w* = 1/[σ^2^(*F*_o_^2^) + (0.0701*P*)^2^ + 6.4307*P*] where *P* = (*F*_o_^2^ + 2*F*_c_^2^)/3
*S* = 1.15	(Δ/σ)_max_ < 0.001
2912 reflections	Δρ_max_ = 0.36 e Å^−^^3^
196 parameters	Δρ_min_ = −0.34 e Å^−^^3^
Primary atom site location: structure-invariant direct methods	Extinction correction: none

### Fractional atomic coordinates and isotropic or equivalent isotropic displacement parameters (Å^2^)

**Table:**

	*x*	*y*	*z*	*U*_iso_*/*U*_eq_	
O1	0.44630 (12)	0.4071 (3)	0.5431 (5)	0.0292 (8)	
O2	0.46014 (12)	0.6355 (3)	0.6148 (5)	0.0292 (8)	
H2	0.4875	0.6225	0.5606	0.044*	
C1	0.38217 (16)	0.5270 (4)	0.7216 (6)	0.0185 (9)	
H1	0.3818	0.6204	0.7818	0.022*	
C2	0.38377 (16)	0.4141 (4)	0.8808 (6)	0.0196 (9)	
H2A	0.3872	0.3212	0.8215	0.024*	
C3	0.33177 (17)	0.4167 (5)	0.9728 (6)	0.0264 (10)	
H3	0.3321	0.3814	1.0967	0.032*	
C4	0.28418 (16)	0.4675 (4)	0.8859 (5)	0.0169 (9)	
C5	0.28100 (17)	0.5288 (5)	0.6930 (6)	0.0254 (10)	
H5A	0.2737	0.6290	0.7038	0.030*	
H5B	0.2501	0.4872	0.6175	0.030*	
C6	0.33030 (16)	0.5119 (5)	0.5815 (6)	0.0201 (9)	
H6	0.3296	0.4172	0.5249	0.024*	
C7	0.43253 (17)	0.5160 (5)	0.6182 (6)	0.0222 (10)	
C8	0.43195 (18)	0.4343 (5)	1.0310 (6)	0.0289 (11)	
H8A	0.4325	0.5299	1.0768	0.043*	
H8B	0.4649	0.4149	0.9748	0.043*	
H8C	0.4288	0.3708	1.1361	0.043*	
C9	0.32793 (19)	0.6213 (5)	0.4201 (6)	0.0295 (11)	
H9A	0.2949	0.6096	0.3377	0.044*	
H9B	0.3583	0.6084	0.3473	0.044*	
H9C	0.3291	0.7146	0.4742	0.044*	
C10	0.23456 (15)	0.4701 (4)	0.9897 (5)	0.0155 (8)	
C11	0.22526 (16)	0.3688 (4)	1.1286 (6)	0.0188 (9)	
H11	0.2501	0.2961	1.1543	0.023*	
C12	0.18002 (16)	0.3744 (4)	1.2283 (6)	0.0193 (9)	
H12	0.1754	0.3056	1.3199	0.023*	
C13	0.14075 (16)	0.4812 (4)	1.1952 (6)	0.0184 (9)	
C14	0.15024 (17)	0.5810 (5)	1.0570 (6)	0.0210 (9)	
H14	0.1253	0.6536	1.0310	0.025*	
C15	0.19560 (16)	0.5761 (5)	0.9565 (6)	0.0208 (9)	
H15	0.2002	0.6450	0.8649	0.025*	
C16	0.09164 (16)	0.4845 (4)	1.3099 (5)	0.0180 (9)	
C17	0.05339 (17)	0.6074 (5)	1.2524 (6)	0.0243 (10)	
H17A	0.0725	0.6951	1.2741	0.036*	
H17B	0.0232	0.6049	1.3279	0.036*	
H17C	0.0405	0.5994	1.1190	0.036*	
C18	0.05936 (17)	0.3457 (5)	1.2774 (6)	0.0254 (10)	
H18A	0.0473	0.3358	1.1436	0.038*	
H18B	0.0286	0.3478	1.3500	0.038*	
H18C	0.0821	0.2672	1.3184	0.038*	
C19	0.11093 (18)	0.4982 (5)	1.5252 (6)	0.0234 (10)	
H19A	0.1315	0.4160	1.5670	0.035*	
H19B	0.0801	0.5065	1.5967	0.035*	
H19C	0.1332	0.5808	1.5458	0.035*	

### Atomic displacement parameters (Å^2^)

**Table:**

	*U*^11^	*U*^22^	*U*^33^	*U*^12^	*U*^13^	*U*^23^
O1	0.0299 (17)	0.0191 (17)	0.0421 (19)	−0.0048 (14)	0.0203 (14)	−0.0079 (14)
O2	0.0269 (18)	0.0221 (17)	0.0423 (19)	−0.0054 (14)	0.0215 (15)	−0.0045 (14)
C1	0.021 (2)	0.015 (2)	0.021 (2)	−0.0007 (17)	0.0055 (17)	−0.0055 (17)
C2	0.020 (2)	0.017 (2)	0.023 (2)	0.0000 (18)	0.0073 (17)	0.0010 (17)
C3	0.026 (2)	0.026 (2)	0.029 (2)	0.004 (2)	0.0145 (19)	0.0088 (19)
C4	0.020 (2)	0.012 (2)	0.019 (2)	−0.0038 (17)	0.0034 (16)	−0.0011 (16)
C5	0.019 (2)	0.036 (3)	0.022 (2)	−0.007 (2)	0.0055 (17)	0.0002 (19)
C6	0.023 (2)	0.020 (2)	0.018 (2)	−0.0015 (18)	0.0058 (17)	−0.0021 (17)
C7	0.024 (2)	0.020 (2)	0.023 (2)	−0.0027 (19)	0.0079 (18)	−0.0017 (18)
C8	0.028 (2)	0.027 (3)	0.032 (2)	−0.004 (2)	0.0022 (19)	0.005 (2)
C9	0.034 (3)	0.031 (3)	0.025 (2)	−0.001 (2)	0.0059 (19)	0.0080 (19)
C10	0.0137 (19)	0.016 (2)	0.0166 (19)	−0.0021 (17)	0.0015 (15)	−0.0039 (16)
C11	0.018 (2)	0.016 (2)	0.022 (2)	0.0001 (17)	0.0020 (16)	0.0003 (16)
C12	0.021 (2)	0.018 (2)	0.020 (2)	−0.0021 (17)	0.0074 (16)	0.0030 (16)
C13	0.017 (2)	0.016 (2)	0.021 (2)	−0.0036 (17)	−0.0008 (16)	−0.0031 (17)
C14	0.022 (2)	0.019 (2)	0.022 (2)	0.0035 (18)	0.0040 (17)	0.0032 (17)
C15	0.021 (2)	0.019 (2)	0.021 (2)	−0.0008 (18)	−0.0009 (17)	0.0040 (17)
C16	0.019 (2)	0.018 (2)	0.018 (2)	0.0020 (17)	0.0046 (16)	0.0004 (16)
C17	0.020 (2)	0.027 (3)	0.026 (2)	0.0053 (19)	0.0032 (17)	0.0034 (19)
C18	0.020 (2)	0.026 (2)	0.032 (2)	−0.0058 (19)	0.0082 (18)	−0.0069 (19)
C19	0.023 (2)	0.023 (2)	0.024 (2)	0.0021 (19)	0.0011 (17)	−0.0031 (18)

### Geometric parameters (Å, °)

**Table:**

O1—C7	1.222 (5)	C2—H2A	0.9800
O2—C7	1.325 (5)	C3—H3	0.9300
C1—C7	1.510 (5)	C5—H5A	0.9700
C1—C2	1.544 (6)	C5—H5B	0.9700
C1—C6	1.545 (6)	C6—H6	0.9800
C2—C3	1.500 (5)	C8—H8A	0.9600
C2—C8	1.523 (6)	C8—H8B	0.9600
C3—C4	1.361 (6)	C8—H8C	0.9600
C4—C5	1.466 (6)	C9—H9A	0.9600
C4—C10	1.493 (5)	C9—H9B	0.9600
C5—C6	1.525 (5)	C9—H9C	0.9600
C6—C9	1.531 (6)	C11—H11	0.9300
C10—C15	1.397 (6)	C12—H12	0.9300
C10—C11	1.402 (6)	C14—H14	0.9300
C11—C12	1.382 (5)	C15—H15	0.9300
C12—C13	1.407 (6)	C17—H17A	0.9600
C13—C14	1.390 (6)	C17—H17B	0.9600
C13—C16	1.527 (5)	C17—H17C	0.9600
C14—C15	1.388 (6)	C18—H18A	0.9600
C16—C17	1.530 (6)	C18—H18B	0.9600
C16—C19	1.542 (6)	C18—H18C	0.9600
C16—C18	1.544 (6)	C19—H19A	0.9600
O2—H2	0.8200	C19—H19B	0.9600
C1—H1	0.9800	C19—H19C	0.9600
			
C7—C1—C2	109.7 (3)	C2—C3—H3	118.0
C7—C1—C6	111.4 (3)	C4—C5—H5A	108.0
C2—C1—C6	110.7 (3)	C6—C5—H5A	108.0
C3—C2—C8	110.5 (4)	C4—C5—H5B	108.0
C3—C2—C1	109.8 (3)	C6—C5—H5B	108.0
C8—C2—C1	112.0 (3)	H5A—C5—H5B	107.2
C4—C3—C2	124.0 (4)	C2—C8—H8A	109.5
C3—C4—C5	121.1 (4)	C2—C8—H8B	109.5
C3—C4—C10	120.6 (4)	C2—C8—H8C	109.5
C5—C4—C10	118.2 (4)	H8A—C8—H8B	109.5
C4—C5—C6	117.4 (4)	H8A—C8—H8C	109.5
C5—C6—C9	109.6 (4)	H8B—C8—H8C	109.5
C5—C6—C1	108.9 (3)	C6—C9—H9A	109.5
C9—C6—C1	112.0 (3)	C6—C9—H9B	109.5
C5—C6—H6	108.8	C6—C9—H9C	109.5
C9—C6—H6	108.8	H9A—C9—H9B	109.5
C1—C6—H6	108.8	H9A—C9—H9C	109.5
O1—C7—O2	123.1 (4)	H9B—C9—H9C	109.5
O1—C7—C1	123.1 (4)	C12—C11—H11	119.2
O2—C7—C1	113.8 (3)	C10—C11—H11	119.2
C15—C10—C11	116.6 (4)	C11—C12—H12	119.0
C15—C10—C4	121.5 (4)	C13—C12—H12	119.0
C11—C10—C4	122.0 (4)	C15—C14—H14	118.9
C12—C11—C10	121.6 (4)	C13—C14—H14	118.9
C11—C12—C13	121.9 (4)	C14—C15—H15	119.2
C14—C13—C12	116.1 (4)	C10—C15—H15	119.2
C14—C13—C16	123.6 (4)	C16—C17—H17A	109.5
C12—C13—C16	120.2 (4)	C16—C17—H17B	109.5
C15—C14—C13	122.3 (4)	H17A—C17—H17B	109.5
C14—C15—C10	121.5 (4)	C16—C17—H17C	109.5
C13—C16—C17	112.5 (3)	H17A—C17—H17C	109.5
C13—C16—C19	109.4 (3)	H17B—C17—H17C	109.5
C17—C16—C19	108.5 (3)	C16—C18—H18A	109.5
C13—C16—C18	109.6 (3)	C16—C18—H18B	109.5
C17—C16—C18	108.0 (3)	H18A—C18—H18B	109.5
C19—C16—C18	108.8 (3)	C16—C18—H18C	109.5
C7—O2—H2	109.5	H18A—C18—H18C	109.5
C7—C1—H1	108.3	H18B—C18—H18C	109.5
C2—C1—H1	108.3	C16—C19—H19A	109.5
C6—C1—H1	108.3	C16—C19—H19B	109.5
C3—C2—H2A	108.1	H19A—C19—H19B	109.5
C8—C2—H2A	108.1	C16—C19—H19C	109.5
C1—C2—H2A	108.1	H19A—C19—H19C	109.5
C4—C3—H3	118.0	H19B—C19—H19C	109.5
			
C2—C3—C4—C5	−2.0 (7)	C3—C4—C10—C15	147.3 (4)
C3—C4—C5—C6	9.7 (6)	C5—C4—C10—C15	−29.1 (6)
C7—C1—C2—C3	−175.2 (4)	C3—C4—C10—C11	−31.1 (6)
C7—C1—C6—C5	−178.2 (4)	C5—C4—C10—C11	152.6 (4)
C2—C1—C6—C9	−179.2 (3)	C15—C10—C11—C12	−0.4 (6)
C4—C5—C6—C9	−160.8 (4)	C4—C10—C11—C12	178.0 (4)
C6—C1—C2—C8	−175.0 (3)	C10—C11—C12—C13	0.4 (6)
C8—C2—C3—C4	147.4 (4)	C11—C12—C13—C14	−0.3 (6)
C6—C1—C2—C3	−51.8 (4)	C11—C12—C13—C16	−179.4 (4)
C7—C1—C2—C8	61.6 (4)	C12—C13—C14—C15	0.2 (6)
C1—C2—C3—C4	23.4 (6)	C16—C13—C14—C15	179.3 (4)
C2—C3—C4—C10	−178.2 (4)	C13—C14—C15—C10	−0.3 (7)
C10—C4—C5—C6	−174.0 (4)	C11—C10—C15—C14	0.4 (6)
C4—C5—C6—C1	−38.0 (5)	C4—C10—C15—C14	−178.1 (4)
C2—C1—C6—C5	59.4 (4)	C14—C13—C16—C17	1.5 (6)
C7—C1—C6—C9	−56.8 (5)	C12—C13—C16—C17	−179.5 (4)
C2—C1—C7—O1	55.7 (6)	C14—C13—C16—C19	−119.2 (4)
C6—C1—C7—O1	−67.3 (6)	C12—C13—C16—C19	59.9 (5)
C2—C1—C7—O2	−124.9 (4)	C14—C13—C16—C18	121.7 (4)
C6—C1—C7—O2	112.0 (4)	C12—C13—C16—C18	−59.3 (5)

### Hydrogen-bond geometry (Å, °)

**Table:**

*D*—H···*A*	*D*—H	H···*A*	*D*···*A*	*D*—H···*A*
O2—H2···O1^i^	0.82	1.88	2.702 (4)	175

Symmetry codes: (i) −*x*+1, −*y*+1, −*z*+1.

## References

[bb1] Altomare, A., Cascarano, G., Giacovazzo, C., Guagliardi, A., Burla, M. C., Polidori, G. & Camalli, M. (1994). *J. Appl. Cryst.* **27**, 435.

[bb2] Bernstein, J., Davis, R., Shimoni, L. & Chang, N.-L. (1995). *Angew. Chem. Int. Ed. Engl.* **34**, 1555–1573.

[bb3] Bruker (2005). *SADABS*, *SAINT* and *APEX2* Bruker AXS Inc., Madison, Wisconsin, USA.

[bb4] Molecular Structure Corporation (1997). *TEXSAN* MSC, The Woodlands, Texas, USA.

[bb5] Sheldrick, G. M. (2008). *Acta Cryst.* A**64**, 112–122. 10.1107/S0108767307043930 18156677

[bb6] Spek, A. L. (2003). *J. Appl. Cryst.* **36**, 7–13.

[bb7] Xie, S., Hou, Y., Meyers, C. Y. & Robinson, P. D. (2002). *Acta Cryst.* E**58**, o1460–o1462. 10.1107/s0108270102000252 11870313

[bb8] Xie, S., Kenny, C. & Robinson, P. D. (2007*a*). *Acta Cryst.* E**63**, o3897.

[bb9] Xie, S., Kenny, C. & Robinson, P. D. (2007*b*). *Acta Cryst.* E**63**, o1660–o1662.

[bb10] Xie, S., Meyers, C. Y. & Robinson, P. D. (2004). *Acta Cryst.* E**60**, o1362–o1364.

